# Cyst identification in retinal optical coherence tomography images using hidden Markov model

**DOI:** 10.1038/s41598-022-27243-2

**Published:** 2023-01-02

**Authors:** Niloofarsadat Mousavi, Maryam Monemian, Parisa Ghaderi Daneshmand, Mohammad Mirmohammadsadeghi, Maryam Zekri, Hossein Rabbani

**Affiliations:** 1 Department of Electrical and Computer Engineering, Isfahan University of Technology, Isfahan, Iran; 2 Medical Image and Signal Processing Research Center, Isfahan University of Medical Sciences, Isfahan, Iran; 3 Negah Hospital, Tehran, Iran

**Keywords:** Computer science, Biomedical engineering

## Abstract

Optical Coherence Tomography (OCT) is a useful imaging modality facilitating the capturing process from retinal layers. In the salient diseases of retina, cysts are formed in retinal layers. Therefore, the identification of cysts in the retinal layers is of great importance. In this paper, a new method is proposed for the rapid detection of cystic OCT B-scans. In the proposed method, a Hidden Markov Model (HMM) is used for mathematically modelling the existence of cyst. In fact, the existence of cyst in the image can be considered as a hidden state. Since the existence of cyst in an OCT B-scan depends on the existence of cyst in the previous B-scans, HMM is an appropriate tool for modelling this process. In the first phase, a number of features are extracted which are Harris, KAZE, HOG, SURF, FAST, Min-Eigen and feature extracted by deep AlexNet. It is shown that the feature with the best discriminating power is the feature extracted by AlexNet. The features extracted in the first phase are used as observation vectors to estimate the HMM parameters. The evaluation results show the improved performance of HMM in terms of accuracy.

## Introduction

One of the most important organs of body is retina. Retina is a layered and light-scattering structure that each layer has its own characteristics. In some important retinal diseases, the properties of layers change or some abnormalities are formed between or inside layers. Age-related Macular Degeneration (AMD) and Diabetic Macular Edema (DME) are from salient retinal diseases which may damage retina and even lead to blindness. The formation of cysts between or inside retinal layers is from the most important manifestations of such diseases. These cysts can have different sizes and shapes which make the process of detection and segmentation difficult. Therefore, the identification of the cysts in such images is of significant importance to control the progressive trend of diseases^1,2^.

Optical Coherence Tomography (OCT) is a comparatively new modality designed for imaging from light-scattering organs like retina. OCT is a non-invasive technology which can provide important information from retinal layers for ophthalmologists. OCT images captured from each patient include a large volume of information the interpretation of which is a time-consuming and difficult process. Thus, to propose new methods for automatically processing of OCT images is of considerable importance^1–4^.

With respect to the automatic methods for identifying cysts in retinal OCT images, a number of works have been proposed^5–26^. Table 1 presents a summary of existing research works for cyst identification and segmentation. It is possible to categorize the existing works according to the approaches utilized for the mentioned purpose. The main approaches are artificial intelligence, graph theory, level set and hybrid methods.

**Table 1:** A summary of existing methods for cyst identification and segmentation.

Research work	Basis of the method	Purpose
^5^	Bilateral filtering, thresholding	Cyst segmentation
^6^	Dictionary learning	Volumetric cyst segmentation
^7,16^	Deep learning (CNN)	Intra retinal cyst segmentation
^8^	Deep learning	Cyst segmentation
^11^	Graph search/Graph cut	Cyst (SEAD) segmentation
^13,22^	Fuzzy level set method	Volumetric cyst segmentation
^14^	Machine learning, Graph theory	Retinal layers and cyst segmentation
^15^	Deep learning (CNN)	Retinal layers and cyst segmentation
^18^	Generating binary and heat maps	Cyst identification
^27^	Deep learning (U-Net), SE blocks	Cyst segmentation, classification of cases to AMD and normal
^28^	Graph theory	Cyst segmentation
^29–31^	Deep learning (CNN)	OCT image classification based on type of disease

In Ref.^8^ a cyst segmentation method is proposed working based on the selective enhancement of cysts. This process is performed by a Convolutional Neural Network (CNN). The construction of Generalized Motion Patterns (GMP) helps to the selective enhancement of cysts. Then, only cysts are enhanced using CNN and a newly defined function. The output of CNN is a probability map where the pixels which belong to cysts have higher probabilities than others^8^.

In Ref.^10^, cysts are detected using a comprehensive analysis of various features. At first, the region-of-interest which is between Inner Limiting Membrane (ILM) and Retinal Pigment Epithelium (RPE) is determined using Dijkstra algorithm. Then, the method analyzes square windows in the image. A large set of features are defined and verified for each window and then several feature selectors are utilized to determine an optimal set of the first features set. Then, the optimal features are utilized for training classifiers.

In Ref.^11^ an approach for the segmentation of cysts in OCT B-scans is proposed which works based on graph theory. Firstly, a classifier is used to produce the probabilities of belonging to the image abnormalities for voxels and the voxels with the highest probabilities are found. Then, graph-search method is used to segment retinal layers. In the next stage, the segmentation of Symptomatic Exudate-Associated Derangement (SEAD) is performed with graph-cut method which has a good performance in the object segmentation.

For the volumetric segmentation of cysts in retinal OCT images, a method based on fuzzy level set is proposed in Ref.^13^. Using the fact that cysts are darker than retinal tissues, the cysts are firstly detected by fuzzy C-means method. Then, level set method is executed on OCT B-scans and C-scans to detect cysts.

In order to 3-dimensionally segment cysts in the OCT B-scan, a fuzzy level set method is proposed in Ref.^22^. The boundaries of cysts in OCT B-scans and C-scans are identified using a fuzzy level set method. These boundaries which are obtained from different types of OCT scan are combined to provide a volumetric segmentation for the cysts.

A method for automatic segmentation of cysts is proposed in Ref.^27^. The method works based on a deep learning approach combined with Squeeze-and-Excitation (SE) blocks. The role of SE blocks is to determine the degree of importance of each feature map acquired by U-Net and remove the feature maps with less importance. After segmentation of cysts, the method is capable of classifying the image as an AMD or a normal case.

In Ref.^28^ a method is proposed for the segmentation of cysts which works based on graph theory and designing a cost function. In the design of the cost function, the general properties of the image and the visual characteristics of cysts are carefully considered. In order to differentiate the dark regions which belong to healthy tissues and cysts, a sheet-ness measure is introduced in the computation of which the Hessian matrix and its Frobenius norm should be calculated.

In Ref.^29^ a Computer Aided Diagnosis (CAD) system is proposed to classify normal, AMD and DME cases with the analysis of OCT B-scans. This method works based on deep learning approach and introduces a novel cost function for rapid learning of features. The main idea is to apply CNN on the sub-images in multiple scales. In Ref.^30^ a deep CNN is proposed for the classification of important retinal diseases including DME, AMD, DR, choroidal neovascularization, macular hole, and central serous retinopathy. In order to extract the features with higher discrimination power, a unified framework is used in Ref.^30^ where fine-tuned pre-trained CNNs are integrated with a new attention-based procedure. In Ref.^31^ a lesion-based CNN is proposed for the classification of OCT images into four classes including drusen, DME, CNV and normal. Firstly, a lesion detection network is designed which can detect different kinds of lesions and produce attention maps. Then, these attention maps are utilized to weigh convolutional feature maps. In fact, the information of lesion-related regions helps to improve the classification accuracy. A CNN-based method for segmenting cysts is suggested in Ref.^7^. After de-noising and layer segmentation, a Fully Convolutional Network (FCN) captures local and global features of image to segment the cysts. A method for the segmentation of cysts in the retinal OCT images of several vendors is proposed in Ref.^32^. This method utilizes several CNNs which are separately trained at different scales to determine whether or not each pixel belongs to cyst. Then, the results of different scales are appropriately combined. Another CNN-based method for segmenting retinal layers and cysts is suggested in Ref.^33^. The method firstly segments the layers through a graph-based approach. The results of layer segmentation are given to a CNN for cyst segmentation regardless of vendor. In addition, a CNN-based method for the same purpose is proposed in Ref.^34^ where a new separable encoder-decoder approach is designed to reduce the task complexity.

In this paper, a new method is proposed for detecting OCT B-scans including cysts with acceptable speed and accuracy. It should be mentioned that the most important application of the proposed method is to find the B-scans including cysts among a large number of B-scans for the ophthalmologist. In fact, in this application there is no need to determine the location or the boundaries of cysts and it is only sufficient to determine the cystic images. For this purpose, it is necessary to find the properties of the B-scans which include cysts. The proposed method firstly finds the most appropriate feature which can discriminate the OCT images including cysts from the ones without cysts. Support Vector Machine (SVM) and KNN classifiers is utilized here to choose the best feature. Then, a HMM is used to identify the status of a B-scan as including or not including cysts. The best extracted features are utilized in the HMM to estimate the parameters of the model. The main contributions of the proposed method in this paper are summarized as follows.

- Using HMM to identify whether or not a B-scan includes cysts.
- To find the best feature for discriminating the B-scans containing cysts from the ones not containing any cyst through SVM and KNN classifiers.
- To determine the capability of different local features for the detection of cystic images.
- To determine the status of an OCT B-scan from cyst point of view based on the status of previous B-scan.

The rest of paper is organized as follows. The proposed method is described in details in “Methods”. “Performance evalution” includes the numerical results obtained from the performance evaluation of the proposed method. “Discussion” includes a detailed discussion about the model results. Finally, the concluding remarks are presented in “Conclusion”.

## Methods

In this section, the proposed method is explained in details. The proposed method includes pre-processing, local feature extraction, choosing the best discriminating feature, and training HMM phases which are verified in the following.

### Preliminaries

In this section, the required explanations about the proposed method are presented. The main part is to introduce Hidden Markov Model (HMM). HMM is a statistical model in which there is a stochastic process which is not observable. This stochastic process is verified only by the help of another stochastic process which is a sequence of observable states. The main elements of an HMM consist of the number of states, the state in the current time which depends on the state in the previous state, and the observations probabilities distribution^32^.

**Figure 1:**
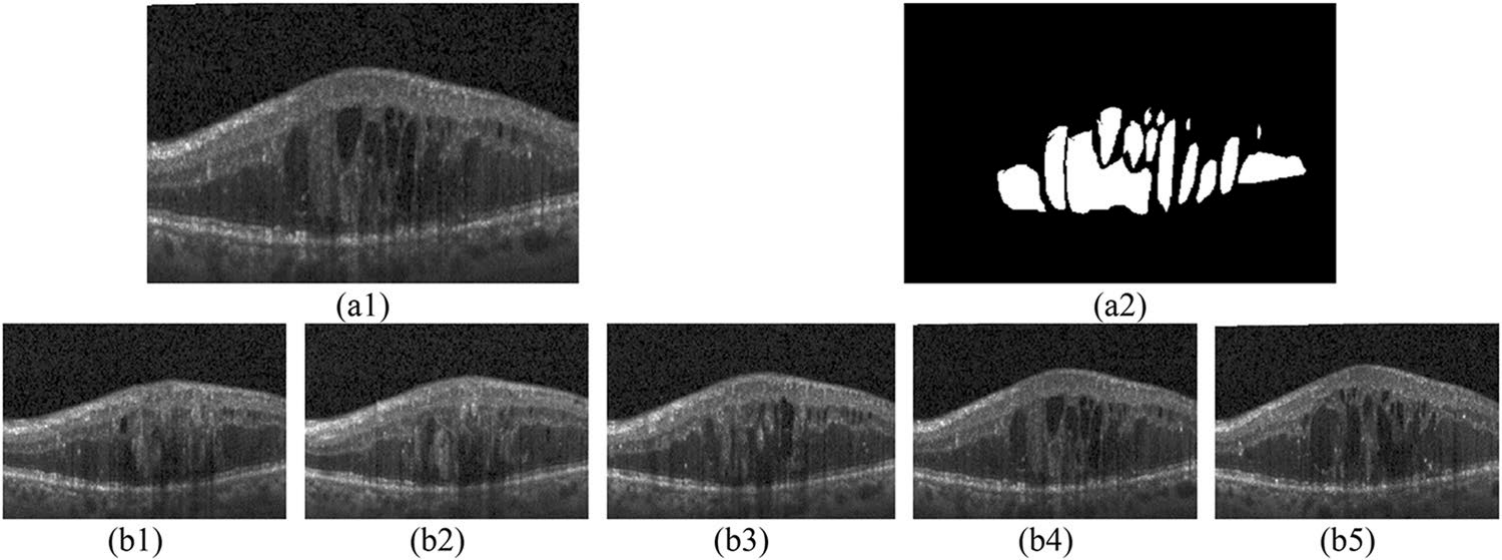
(**a1**) An OCT B-scan containing cysts, and (**a2**) its cysts presented with white regions, (**b1**)–(**b5**) a series of consecutive B-scans which belong to one specific subject.

Figure 1 presents OCT B-scans which may consist of cysts. The image in part (a1) is an OCT B-scan including cysts. Cysts are dark regions located intra retinal layers or stick to the layer boundaries. They are presented with white color in part (a2). The images in parts (b1) to (b5) are the consecutive B-scans with numbers 21 to 25 in one specific subject. It can be observed that the existence of cyst in one B-scan depends on the existence of cyst in the previous B-scan. In fact, if one B-scan includes cyst, the next B-scan consists of cyst with high probability. The existence of cyst in one B-scan can be inferred as a hidden state which provides some distinguishing features for the B-scan. Therefore, it sounds reasonable to model the existence of cyst in the B-scan with HMM.

### Datasets

The datasets used in this research work contains OCT B-scans captured from retina. The first dataset called Dataset 1 contains the images which are captured by SD-OCT Heidelberg device. This device is capable of capturing 40,000 A-scans per second. The dataset has been prepared in Noor Ophthalmology hospital and consists of data from 50 healthy persons, 48 dry AMD patients and 50 DME patients. The number of OCT B-scans is not the same for all persons and can be one of the values of 19, 25, 31, and 61. The OCT B-scans of this dataset are gray-scale. The number of OCT B-scans containing cysts is equal to 1360. Also, 1700 OCT B-scans do not include any cyst. The dataset is available in https://misp.mui.ac.ir.

The second dataset called Dataset 2 is publicly online at http://people.duke.edu/~sf59/Chiu_BOE_2014_dataset.htm. This dataset consists of 110 OCT B-scans from 10 patients with severe DME pathology^33^. The device used for capturing images is Heidelberg which uses 61 lines volume protocol. In fact, for each subject, 61 OCT B-scans are captured. However, the expert annotations are available only for 11 OCT B-scans from all the B-scans of each subject.

In order to use the images of both datasets, it is necessary to put images into two training and testing subsets. From all OCT B-scans, 80% and 20% are used for training and testing, respectively.

### Pre-processing

In this phase, the margins of OCT B-scans which do not contain useful information are removed from the images. However, since retinal layers are very close to top or bottom margin in some OCT B-scans, it may not be possible to remove a large number of rows from top or bottom of image. It should be mentioned that the number of columns which are removed from the left and right parts of an OCT B-scan is equal to 13 and 3, respectively. Also, 20 and 10 rows from top and bottom of the OCT B-scan are omitted from the image. Since one main purpose is to propose simple and rapid algorithm, only margin elimination is performed in this phase. Also, since feature extraction methods are robust to noise, no de-noising method is applied on the images in this phase.

### Local feature extraction

In this phase, the local features are extracted. The features which are extracted are explained in the following.

#### Histogram of oriented gradient (HOG) feature

In order to extract this feature, it is necessary to perform several steps. For each pixel, it is required to compute the gradient vector which consists of magnitude and orientation. In order to compute the gradient of each pixel, two matrices which are $${G}_{x}=\left[\begin{array}{c}-1\\ -2\\ -1\end{array} \begin{array}{c}0\\ 0\\ 0\end{array}\begin{array}{c}1\\ 2\\ 1\end{array}\right]$$ and $${G}_{y}=\left[\begin{array}{c}1\\ 0\\ -1\end{array} \begin{array}{c}2\\ 0\\ 2\end{array} \begin{array}{c}1\\ 0\\ -1\end{array}\right]$$ should be convolved with a 3*3 intensity matrix around the pixel. Let *s*(*i* − 1:*i* + 1, *j* − 1:*j* + 1) denote the intensity values of pixels which are located between rows *i* − 1 to *i* + 1 and columns *j* − 1 to *j* + 1. The magnitude and orientation of gradient for *p*_*i,j*_ are computed via the following equations.

1 $$magnitude(i,j)=\sqrt{{a(i,j)}^{2}+{b(i,j)}^{2}}$$

2 $$\theta \left(i,j\right)=\mathrm{arctan}(\frac{b(i,j)}{a(i,j)})$$

3 $$a\left(i,j\right)={G}_{x}*s(i-1:i+1,j-1:j+1)$$

4 $$b\left(i,j\right)={G}_{y}*s(i-1:i+1,j-1:j+1)$$

Then, the image is divided into d*d cells and for each cell the histogram of gradient orientations is formed. A number of cells are formed a block. The vectors of blocks are connected and make the final HOG feature^34^.

#### Harris feature

Harris algorithm is an edge detector. The main idea in this detector is to detect the points with different intensity values in a local neighborhood. If *h* denotes a small increment in the location, the corners are *x*s which maximize the following function^35^.

5 $$E\left(h\right)=\sum w(x){(I\left(x+h\right)-I(x))}^{2}$$

where in Eq. (5) w(x) denotes a rectangular or Gaussian window. In order to find the maximum of *E*(*h*), a matrix which is called tensor structure should be analyzed. Then, a parameter is computed using the eigenvalues. By making a comparison between the value of this parameter and a threshold, the corners and edges are determined. The value of such a parameter is considered to be equal to 0.01. A Gaussian window with dimensions 5*5 is also considered for *w*(*x*). The standard deviation for such a window is equal to $\frac{5}{3}$.

#### Min-Eigen feature

The algorithm for computing this feature is a developed version of Harris algorithm^36^. In contrast with Harris feature where a parameter is calculated using the eigenvalues, the eigenvalues are utilized in Min-Eigen feature for detecting corners.

#### FAST feature

In the computation of this feature, a circle with the radius of 3 pixels is considered which consists of 16 pixels. The center of this circle is the pixel which we want to determine whether or not it is a corner. The pixels are numbered from 1 to 16 in clock-wise direction. If *N* pixels from 16 pixels are lighter than the sum of intensity value of the center pixel and a threshold, the center pixel is considered as a corner. In addition, if *N* pixels from 16 pixels are darker than the subtraction of a threshold from the intensity value of the center pixel, the center pixel is considered as a corner^37^.

#### SURF feature

For computing SURF feature, the detector works based on Hessian matrix. The purpose is to detect points which maximize the determinant of Hessian matrix which is defined in the following equation.

6 $$\mathrm{det}\left(H\right)={D}_{xx}{D}_{yy}-{(w{D}_{xy})}^{2}$$

where in Eq. (5) ${D}_{xx}$, ${D}_{yy}$, and ${D}_{xy}$ are box filters which are approximates for Gaussian filters^38^.

#### KAZE feature

KAZE algorithm is a developed version of SURF algorithm which utilizes scale space. This algorithm uses non-linear scale space while SURF algorithm finds the features in the linear Gaussian scale space. Three main steps in KAZE algorithm are as follows^39^.

1. To construct a pyramid-like non-linear scale space from the input image
2. To determine the key points by computing the determinant of the Hessian matrix and multi-scale derivatives
3. To compute orientation and form descriptor vector for all key points

#### BRISK feature

The BRISK feature is from binary descriptors which uses scale space. The scale space usually includes four octaves each of which consists of four levels. Octaves are made by reducing sampling rate from the main image. A 9–16 mask is used in BRISK feature. In this algorithm, it is necessary that 9 consecutive pixels from 16 pixels are lighter or darker than the center pixel. This detector is separately applied on all octaves and levels^40^.

### Feature extraction by AlexNet

In this phase, the OCT B-scans are prepared to be appropriate for processing by Alex-Net. It should be noted that the dimensions of OCT B-scans in the dataset are equal to 512*496*1 and the input image for the Alex-Net should be equal to 227*227*3. Therefore, the gray-scale image should be converted to colorful image with the mentioned dimensions. In order to do so, a color is assigned to each image pixel which its intensity value is between 0 and 1. The warmer colors such as red are assigned to the pixels with higher intensity values. Also, the colder colors like blue are assigned to the pixels with lower intensity values. After assigning colors, the image should be resized to 227*227 dimensions. One way is to remove the margins of the image. However, since cysts may be located near the margins of the image, the elimination of margins may lead to the omission of cysts. The better way is to reduce the sampling rate.

Then, the prepared images are fed to AlexNet as inputs. In a research work^44^, DME cases are categorized to two classes which are with cysts and without cysts using transfer learning. It has been shown in^44^ that the best layer for feature extraction is Fully Connected (FC) layer 8. Thus, FC layer 8 is also utilized for feature extraction in this method. These vectors are vectors with the length of 1000 which include values between − 8 and 11.

### Choosing the Best Discriminating Feature

In order to choose the feature which can strongly discriminate the images including cysts from others, SVM and KNN classifiers are utilized. In order to choose the best feature, the extracted feature vectors are converted to input vectors for SVM and KNN. In fact, for each feature extraction method, SVM and KNN are used. The SVM utilized for this purpose consists of two classes the labels of which are with cyst and without cyst, respectively. The OCT B-scans considered for training in the previous phase are used to train SVM and KNN. The SVM is trained with incremental training method. In the incremental training method, SVM is trained with new feature vector without considering previous feature vector.

After training SVM, the OCT B-scans considered for testing are used to evaluate the performance of SVM in the testing phase. In the training phase, the separating hyperplane between two classes is estimated. In the test phase, the extracted feature vectors are considered as x in ${w}^{t}.x+b$ equation where w and b have been obtained in the training phase. Then, the sign of the mentioned term is determined. If the sign is equal to + 1, the image consists of cyst and if it is equal to − 1, the image does not consist of any cyst.

**Figure 2:**
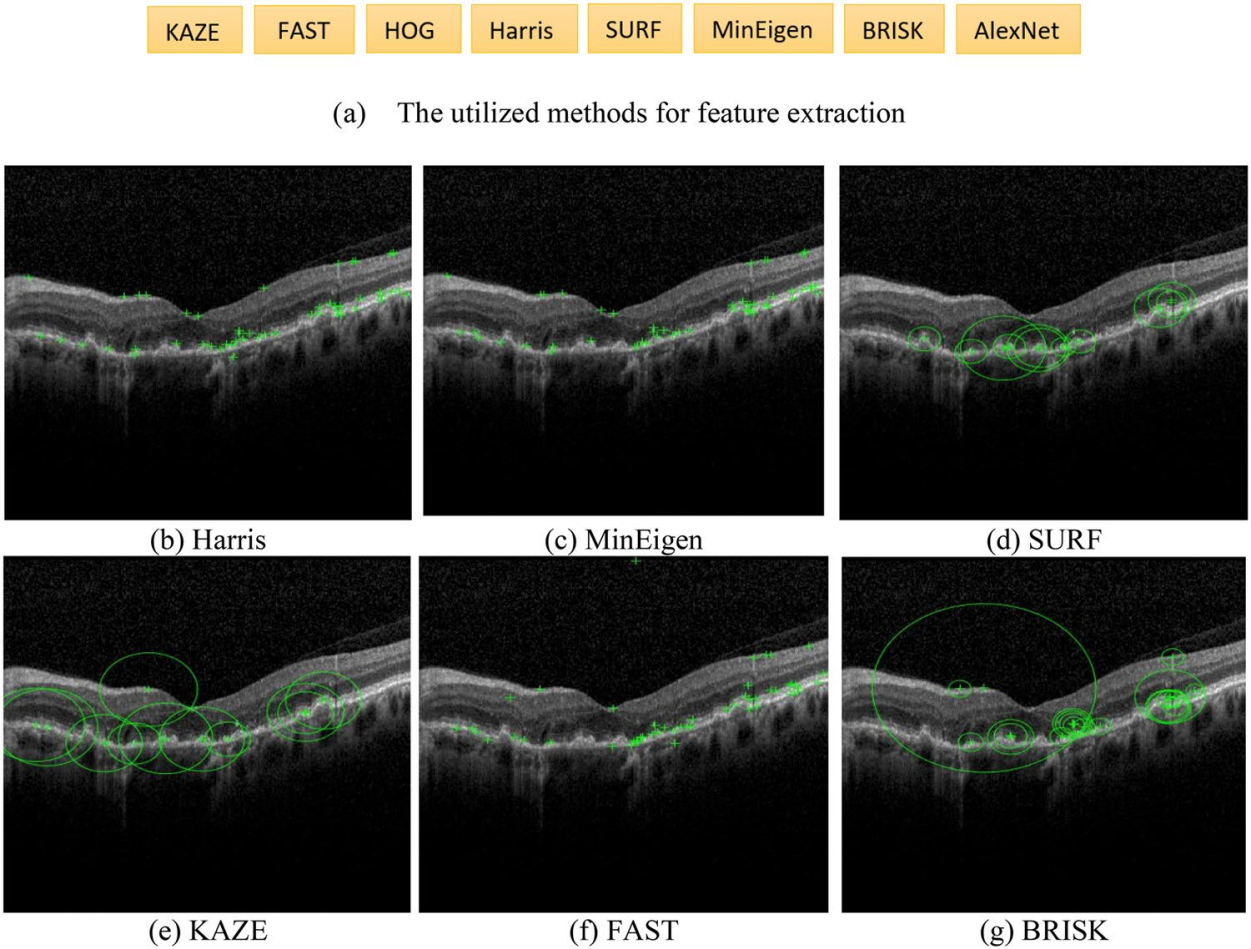
A summary of feature extraction methods and their corresponding key points for cyst identification. The points shown with “+” indicate the strongest points extracted by each method. The green circles are related to the scale at which the features are detected.

The estimated labels with SVM classifier are compared with the expert's labels. Among eight local feature extraction methods, transfer learning method with AlexNet is chosen as the feature extraction method for the proposed algorithm. This feature extraction method is the best feature discriminating the two classes. Figure 2 presents a summary of the methods used for feature extraction. It also shows the key points related to cyst identification extracted by them. Green pluses indicate the locations of points and green circles show the scale.

### Training HMM

In this phase, the process of training HMM is explained. This process starts with choosing the dimensions of HMM. Also, the parameters of model are obtained in the next steps. Then, the appropriate model with proper parameters is used for identifying the images containing cysts.

The dimensions of HMM depend on the dimensions of the observation vector. If the extracted observation vector is 2-dimensional, the HMM should also be 2-dimensional. Thus, if the feature extraction method is KAZE or SURF, the HMM should be 2-dimensional. However, since the feature extraction method is transfer learning which extracts a 1-dimensional vector from each image, the HMM is also 1-dimensional. Moreover, the complexity of 1-dimensional HMM is low. Also, the number of HMMs used in this paper for cyst identification is equal to 1.

Since the existence of cyst in an OCT B-scan depends on the existence of cyst in the previous OCT B-scan, it can be considered as a hidden state. Therefore, the HMM is considered with two hidden states in the proposed method. The first state denoted by *q*_*1*_ is the image including cyst and the second one denoted by *q*_*2*_ is the image without cyst. Moreover, the observations are feature vectors which have been extracted from OCT B-scans. Therefore, the feature vectors extracted by transfer learning method are the observable vectors which their dimensions are equal to 1*1000 for each B-scan.

**Figure 3:**
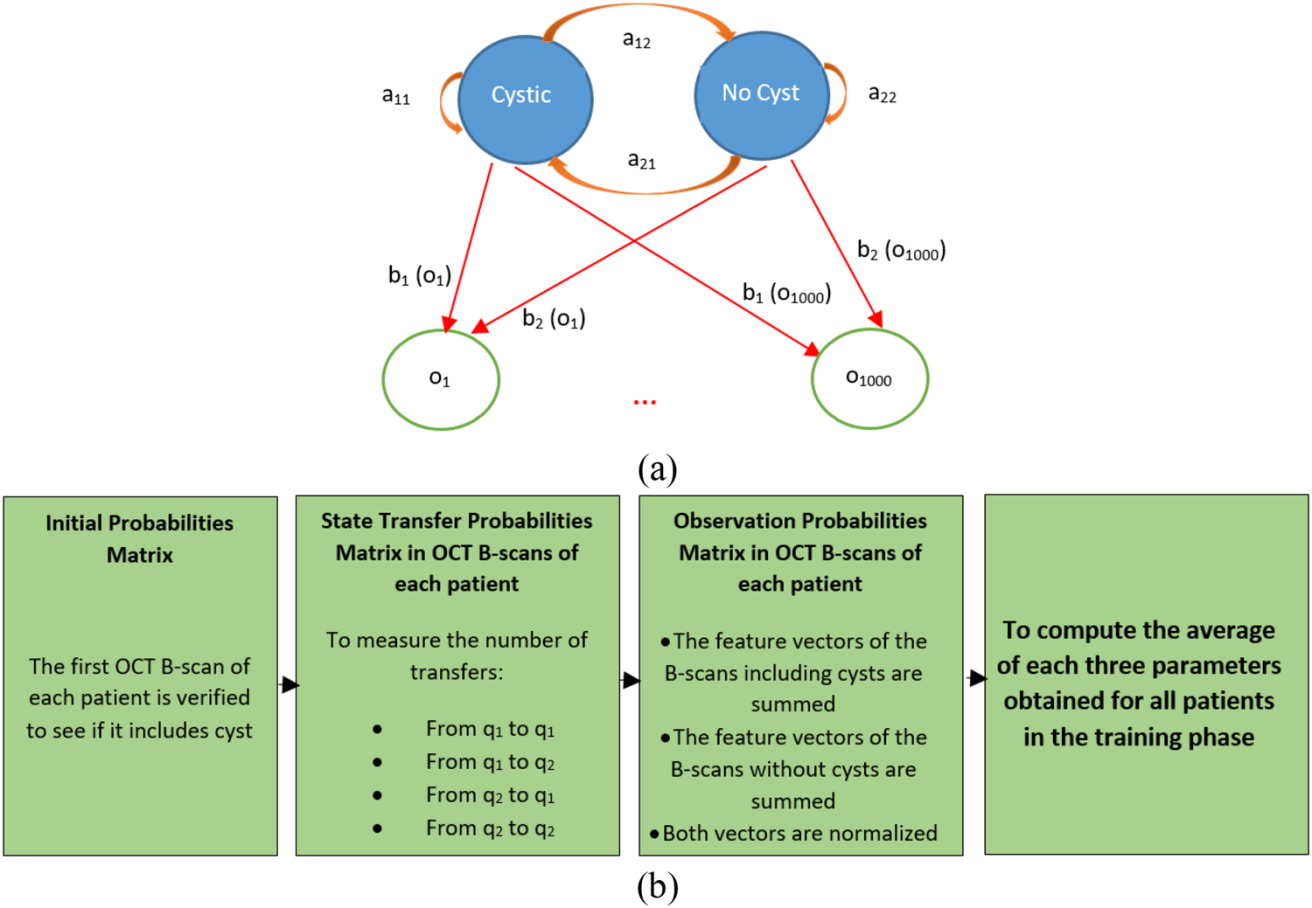
(**a**) The proposed HMM for cyst identification, blue and green circles show states and observations respectively, (**b**) block diagram of training process of the proposed model.

In a series of OCT B-scans which belong to one patient, it is possible to have a cystic image after a cystic or non-cystic image. Thus, there is no constraint for state transfer model and the chosen HMM is ergodic^32^. The proposed HMM is presented in Fig. 3a. In this figure, the states are shown with blue color. Also, the probabilities of state transfer are presented along orange lines. In addition, the probabilities of producing observations by each state are shown along red lines. The number of such probabilities is equal to 1000 for transfer learning method. In fact, there are 1000 observations in this model which are observable with certain probabilities in each state. Let *o*_*i*_ ($1\le i\le 1000$) denote the *i*^th^ observation extracted by AlexNet. Also, ${{b}_{1}(o}_{i})$ ($1\le i\le 1000$) denotes the probability of having *o*_*i*_, if we have a cystic image. Moreover, ${{b}_{2}(o}_{i})$ ($1\le i\le 1000$) denotes the probability of having *o*_*i*_, if we have an image without cyst. In addition, *a*_*11*_ denotes the probability of having a cystic image after a cystic image. Furthermore, *a*_*12*_ denotes the probability of having an image without cyst after a cystic image. Also, *a*_*21*_ denotes the probability of having a cystic image after an image without cyst. Finally, *a*_*22*_ denotes the probability of having an image without cyst after an image without cyst.

According to the above explanations, the model parameters are 2*2 transfer probability distribution matrix, 2*1000 observation producing probability matrix, and 2*1 initial probability matrix. These matrices are denoted with $$A=\left[\begin{array}{c}{a}_{11}\\ {a}_{21}\end{array} \begin{array}{c}{a}_{12}\\ {a}_{22}\end{array}\right]$$, $$B=\left[\begin{array}{c}{{b}_{1}(o}_{1})\\ {{b}_{2}(o}_{1})\end{array}\dots \begin{array}{c}{{b}_{1}(o}_{1000})\\ {{b}_{2}(o}_{1000})\end{array}\right]$$, and $$\pi =\left[\begin{array}{c}{\pi }_{1}\\ {\pi }_{2}\end{array}\right]$$, respectively. They are unknown and should be estimated in the training phase.

In order to compute the model parameters, a method is proposed utilizing the labels of OCT B-scans. The block diagram of this method is presented in Fig. 3b. In this method, the parameters are firstly obtained for the OCT B-scans of each patient. In order to obtain initial probabilities matrix, the first image is verified. If it includes cyst, the initial probabilities matrix is $$\pi =\left[\begin{array}{c}1\\ 0\end{array}\right]$$ and if it does not include any cyst, the initial probabilities matrix is $$\pi =\left[\begin{array}{c}0\\ 1\end{array}\right]$$. Also, in order to compute the transfer probability distribution matrix, the following rules are considered. It should be also considered that the sum of probabilities in each row of A is equal to 1.

${a}_{11}$ =The result of division of the number of transfers from *q*_*1*_ to *q*_*1*_ to the sum of transfers from *q*_*1*_ to *q*_*1*_ and from *q*_*1*_ to *q*_*2*_.

${a}_{12}$ =The result of division of the number of transfers from *q*_*1*_ to *q*_*2*_ to the sum of transfers from *q*_*1*_ to *q*_*2*_ and from *q*_*1*_ to *q*_*1*_.

${a}_{21}$ =The result of division of the number of transfers from *q*_*2*_ to *q*_*1*_ to the sum of transfers from *q*_*2*_ to *q*_*1*_ and from *q*_*2*_ to *q*_*2*_.

${a}_{22}$ = The result of division of the number of transfers from *q*_*2*_ to *q*_*2*_ to the sum of transfers from *q*_*2*_ to *q*_*2*_ and from *q*_*2*_ to *q*_*1*_.

In order to compute the observation probability matrix, the feature vectors of the OCT B-scans including cysts should be added. In fact, each column of a feature vector should be added with the corresponding column in another feature vector. Since it may be possible to find negative values in some feature vectors, it is necessary to firstly compute the absolute value for each vector and then the summation is performed. The reason is that the probability values are also equal or higher than zero. Then, the value of each vector member is divided to the sum of them to make normalization. This vector forms the first row in the observation probability matrix. These computations are performed for the OCT B-scans without cysts and the result vector forms the second row in the observation probability matrix.

Let *N* denote the number of patients used for training the HMM model. Also, let *A*_*i*_, *B*_*i*_ and ${\pi }_{i}$ denote *A*, *B*, and *π* matrix for *i*th patient ($1\le i\le N$), respectively. The parameters of the final model are denoted with $\lambda =({A}_{total},{B}_{total},{\pi }_{total})$ and are obtained via the following equation.

7 $${A}_{total}=\frac{\sum_{i=1}^{N}{A}_{i}}{N}$$

8 $${B}_{total}=\frac{\sum_{i=1}^{N}{B}_{i}}{N}$$

9 $${\pi }_{total}=\frac{\sum_{i=1}^{N}{\pi }_{i}}{N}$$

### Decoding HMM

In order to test the trained HMM, the OCT B-scans of 20% of patients which are not utilized in the training phase are used here. It should be clarified that in this phase, all the images which belong to one subject are given to the HMM. In fact, the proposed HMM determines the status of each image based on its previous image. The method does not classify the image by itself and it at least needs the previous image in the same subject. In this phase, the parameters of the trained HMM, $\lambda =({A}_{total},{B}_{total},{\pi }_{total})$, are available. Also, the extracted observation vectors from the B-scans of test cases are available. These feature vectors have been extracted from AlexNet. Therefore, the HMM and observation vectors are available and it is necessary to find the sequence of states. This is a decoding problem which can be solved with Viterbi algorithm^32^.

At first, the number of observation vectors for each patient in the testing phase is determined. Let *T* denote this parameter. The dimensions of each observation vector are 1*1000. In this step, the absolute values of observation vectors are computed. The number of labels delivered by the decoding algorithm should be equal to *T*. The parameters of Viterbi algorithm are δ and $\varphi$ which are defined with 2*19 dimensions. The first column of δ has the value of ${\updelta }_{1}(i)={\pi }_{i}{b}_{i}({o}_{1})$ where ${o}_{1}$ is the observation vector extracted from the first OCT B-scan. The first column of $\varphi$ has the value of 0. The other values of δ and $\varphi$ are obtained from the following equations.

10 $${\updelta }_{t}\left(j\right)=\mathrm{max}({\updelta }_{t-1}\left(i\right){a}_{ij}){b}_{j}({o}_{t})$$

11 $${\varphi }_{j}\left(t\right)=\mathrm{argmax}({\updelta }_{t-1}\left(i\right){a}_{ij})$$

In order to compute the most probable sequence of states for T B-scans of each patient, the maximum value of the last column of $\updelta$ matrix is found. Let *qP* denote the sequence of states. The row which has the maximum value is considered as the state of *T*^th^ B-scan.

12 $$\left[{i}^{th}\left(T\right),qP\left(T\right)\right]=\mathrm{max}(\updelta (:,T))$$

In order to compute the states 1 to (*T*-1), the value of $\varphi$ in the next states is utilized. The following equation is utilized for this purpose.

13 $$qP\left(t\right)=\varphi (qP\left(t+1\right),t+1)$$

This algorithm is executed for all the patients in the test phase to determine whether their OCT B-scans include cyst. It should be noted that *qP* consists of 1 and 2. The values of "1" and "2" indicate the images containing and not containing cysts, respectively.

## Performance evaluation

In this section, the results of evaluating the proposed model are presented. First of all, the datasets and the required tools are introduced. Then, the evaluation parameters of classifiers are defined. Then, the results of classifying the images by the proposed model are compared with those of SVM classifier, and the results of^33^ and^42^ for categorizing the images.

### Preliminaries

In Dataset 1, the images have been saved with .tiff format. The dimensions of all images are 512*496 pixels. The assignment of labels to the OCT B-scans of each patient has been performed by an ophthalmologist. The dataset includes the information of 148 persons. Since the information of 80% and 20% of cases are used for training and testing phases, the images of 118 and 30 persons are used in the training and testing phases, respectively. The dimensions of images in Dataset 2 are 496*768. The assignment of labels to the OCT B-scans of each patient have been performed by two ophthalmologists.

It is not necessary to apply a noise reduction method on the images. The reason is that the execution of de-noising method reduces the speed of algorithm and does not improve its performance. The software used for processing the images is MATLAB R2018a. Also, the machine vision toolbox is used for local feature extraction and forming feature vector. In addition, AlexNet deep network toolbox is used for feature extraction.

### Evaluation parameters

The most important parameters for evaluating the performance of computerized identification system are accuracy, sensitivity and specificity. These parameters are statistical metrics for the performance evaluation of a binary classification system.

If an OCT B-scan includes cyst, it is considered as a positive case and if an image does not have any cyst, it is considered as a negative case. Therefore, one of the following cases will occur for each image.

- The image is a positive case and it is correctly identified as a positive case (True Positive, TP).
- The image is a negative case and it is correctly identified as a negative one (True Negative, TN).
- The image is a positive case and it is falsely detected as a negative one (False Negative, FN).
- The image is a negative case and it is falsely detected as a positive one (False Positive, FP).

Accuracy parameter is the simplest and the most common metric for measuring the quality of a classifier. Accuracy is a measure of correct identifications in both categories and is computed as follows.

14 $$Accuracy=\frac{TP+TN}{TP+TN+FP+FN}$$

Sensitivity is a metric which determines the capability of classifier in the identification of cystic B-scans. Sensitivity parameter is obtained through the following equation.

15 $$sensitivity=\frac{TP}{TP+FN}$$

Specificity is known as the rate of negative answers. It is computed through the following equation.

16 $$specificity=\frac{TN}{TN+FP}$$

It is clear that in ideal conditions, both sensitivity and specificity have high values.

### Evaluation of different features

In the following, the performance of SVM and KNN classifiers in the classification of OCT B-scans is presented. Table 2 shows the values of sensitivity, specificity and accuracy of SVM and KNN classifiers for different feature extraction methods. As obvious from Table 2, the feature extracted by AlexNet provides the highest values for evaluation metrics. It means that it has the highest power in the discrimination of cystic images from non-cystic ones among other features. Among the local features, HOG has the best performance. The performance of FAST, Harris and MinEigen in the discrimination of cystic from non-cystic images is very weak. Thus, it can be concluded that AlexNet should be selected for feature selection in this dataset.

**Table 2:** Sensitivity, specificity and accuracy of (a) SVM and (b) KNN classifiers for different methods.

Method/parameter	Specificity (%)	Sensitivity (%)		Accuracy (%)
**(a)**				
AlexNet	93	91		92
HOG	93	82		88
KAZE	83	79		82
SURF	82	72		78
BRISK	69	60		65
MinEigen	58	50		55
Harris	60	48		54
FAST	56	48		52
(**b**)				
AlexNet	94	93	92	
HOG	92	88	87	
KAZE	79	72	76	
SURF	64	55	60	
BRISK	64	53	59	
MinEigen	57	51	54	
Harris	53	52	55	
FAST	53	51	52	

**Figure 4:**
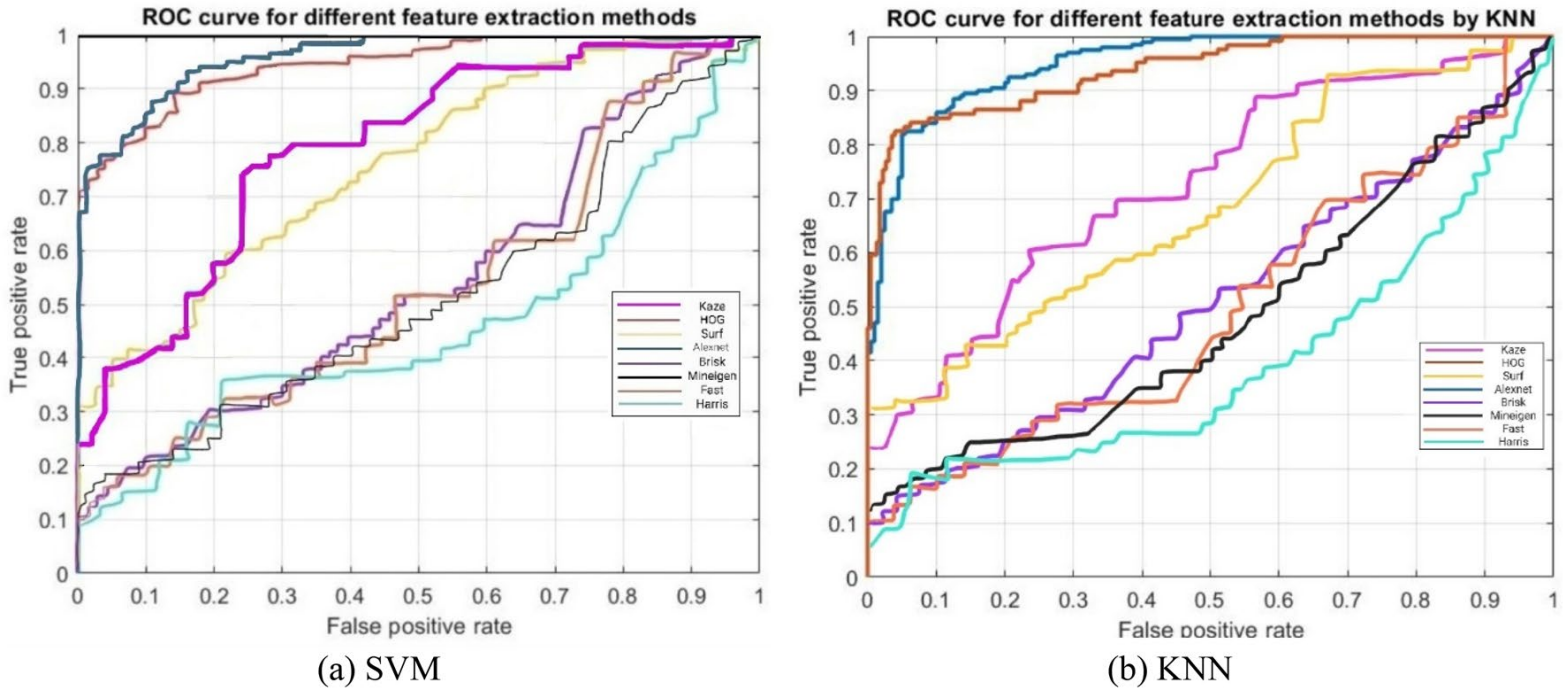
ROC curve for (**a**) SVM and (**b**) KNN classifiers when they are fed with different features.

Figure 4 also presents the ROC (Receiver Operating Characteristic) curve for SVM and KNN classifiers when they are fed by different features. As can be observed in both figures, the feature extracted by AlexNet is the feature providing the best ROC curve for both SVM and KNN classifiers.

The evaluation parameters for the proposed HMM are presented in Table 3. In this table, the results of the proposed HMM are also presented for the case where HOG feature is extracted from the image instead of the feature extracted by AlexNet. As can be observed in the table, the performance of the proposed HMM using the feature extracted by AlexNet is better than the proposed model using HOG feature.

**Table 3:** Sensitivity, specificity and accuracy of CNN and the proposed HMM for two different features in Dataset 1 (a). Sensitivity, specificity and accuracy of the proposed HMM, KR^33^ and CIBICA^42^ in Dataset 2 (b).

Feature/parameter	Specificity	Sensitivity	Accuracy
(**a**)			
HMM with feature extracted by AlexNet	0.97	0.93	0.95
Feature extracted by ResNet	0.97	0.89	0.93
HOG	0.95	0.85	0.91
CNN	0.91	0.89	0.90
(**b**)			
HMM with feature extracted by AlexNet	0.9	0.94	0.92
HMM with feature extracted by ResNet	0.94	0.87	0.9
KR^33^	0.52	0.79	0.7
CIBICA^42^	0.74	0.79	0.75

In addition, we have implemented another pre-trained network for feature extraction which is ResNet. The results of classification obtained from our HMM method for ResNet feature are also presented in Table 3. As can be observed, for Dataset 1, the results for AlexNet are slightly better than the results for Resnet. For Dataset 2, sensitivity and accuracy for AlexNet are also better than the results for Resnet. Generally, for both datasets, the results are comparatively close to each other. In fact, it is possible to utilize ResNet instead of AlexNet for automatic feature extraction and then use HMM with the newly extracted features. Even in the worst case between AlexNet and ResNet, the obtained results are much better than the existing methods such as^33,42^.

In order to compare the results of our HMM model with the state-of-the-art methods, we have chosen the methods of^33,42^. They both utilized Dataset 2 for performance evaluation.

In^33^ a method for segmenting retinal cysts is suggested. The method called KR firstly identifies seven retinal layers and cysts with the help of a kernel regression classification approach. In KR method the local estimates of a function are derived using a nonparametric mathematical model. In defining the kernels, the relative importance of close points are weighed. Firstly, the locations of retinal layers and cysts are determined. Then, a graph theory and dynamic programming framework is used to segment the boundaries of estimated regions.

In^42^ a texture-based method for diagnosis of cystic B-scans has been proposed. In the method called CIBICA, firstly the images are de-noised and the boundary points which provide significant intensity changes in the vertical direction are determined. Then, for each boundary point two windows are considered in the right and left parts. The boundary points which provide considerable intensity changes in the horizontal direction are determined. Then, the number of horizontal boundary points in the mentioned windows are computed. If the number of such points is greater than a threshold, the initial point is considered as a boundary point for a cyst. If an OCT B-scan contains at least one point with such a characteristic, the image is considered as a cystic one.

The results of^33,42^ are presented in part b of Table 3. As clear in the table, the values of sensitivity, specificity and accuracy in the proposed HMM are much higher than the results of^33,42^. In addition, it should be noted that the proposed HMM is capable of providing good results without the need to de-noising operations, while in^33,42^, the image is initially de-noised.

**Figure 5:**
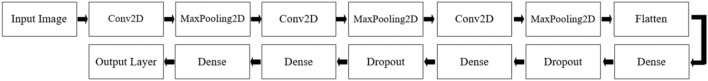
The structure of designed CNN for performance comparison.

The performance of our proposed HMM is also compared with a CNN. In fact, we have implemented a CNN for the classification of images of datasets into cystic and non-cystic ones. The CNN has been applied on the images of the same datasets. In order to train the CNN, the same images which are utilized for training our proposed HMM are used. The structure of the implemented CNN is presented in Fig. 5.

In the CNN model, the 2-D convolution operations have been performed using 64, 32, and 16 filters in the three convolutional layers, respectively. A kernel with size 2*2 and a unit stride sliding window is employed in each convolutional layer. Each convolutional layer is followed by one 2 × 2 Max-pooling layer. In order to train CNN model, the Adam optimizer is used. The learning rate is equal to 0.0001. All Images are resized to 224*224. Rectified-linear-unit (ReLU) is applied as the activation function for the neurons. Table 3 also includes the results of CNN for Dataset 1. It should be noticed that since the number of labeled images in Dataset 2 is very limited and equal to 110 images, it is not possible to test the performance of CNN on this dataset. The reason is that CNN requires a large number of images for training. As can be observed in Table 3, our results are better than those of CNN. However, the main novelty of our method is the development of hidden Markov model which is a strong mathematical model for the application of cyst detection.

The confidence interval for comparing the performance of the proposed HMM with KR-based method^33^ and CIBICA method^42^ (For Dataset 2) is equal to 0.95. Also, the confidence interval for comparing the performance of the proposed HMM versus SVM method (For Dataset 1) is equal to 0.95. These values are computed using t-test statistical method.

**Figure 6:**
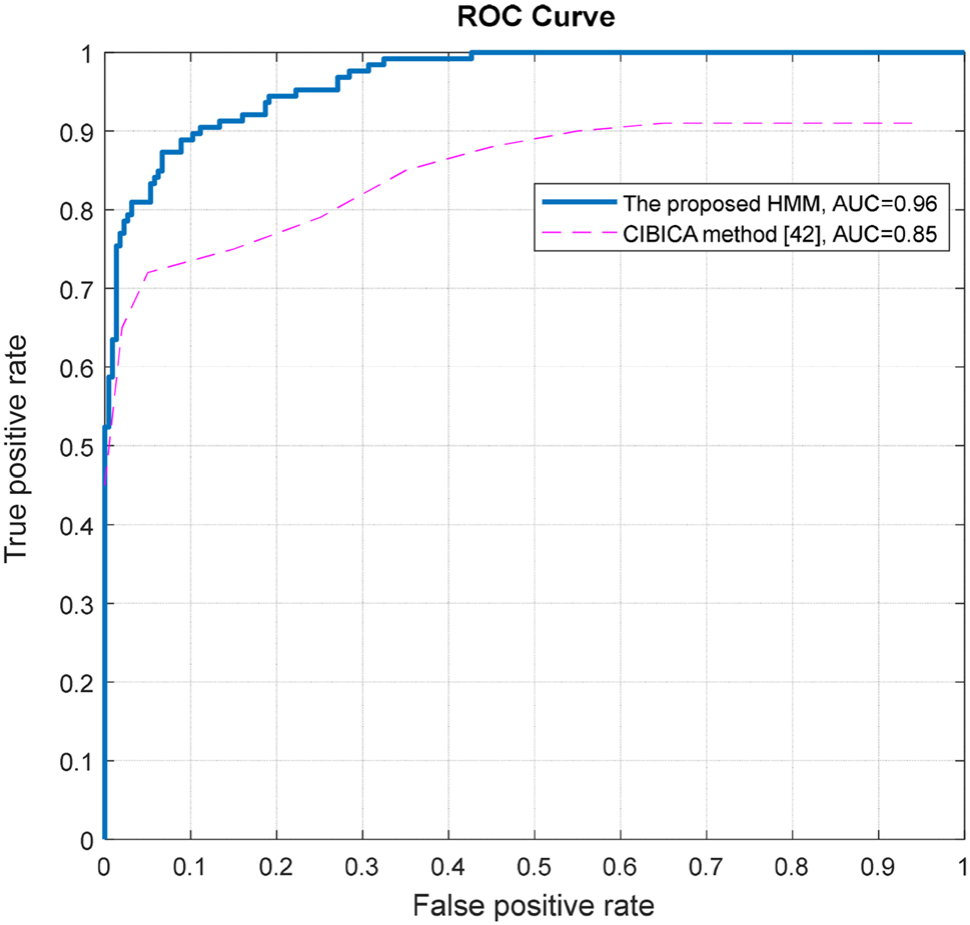
ROC curve for the proposed HMM and the CIBICA method^42^.

Figure 6 presents the ROC curve for the proposed HMM and the CIBICA method of^42^. It is obvious from the figure that the AUC (Area under the Curve) value for the proposed HMM is considerably higher than the CIBICA method.

In order to evaluate the statistical difference between the AUC of the proposed HMM and the CIBICA method of^42^, we utilize DeLong’s test^43^. DeLong’s test verifies the ROC curves of two methods to determine whether or not their AUC values are statically significantly different. The results of this test show that with p-value = 0.0001092 < 0.05, it is confirmed that there is a statistically significantly difference between the proposed HMM and the CIBICA method of^42^. Therefore, our proposed method has a better performance compared to the mentioned method.

**Figure 7:**
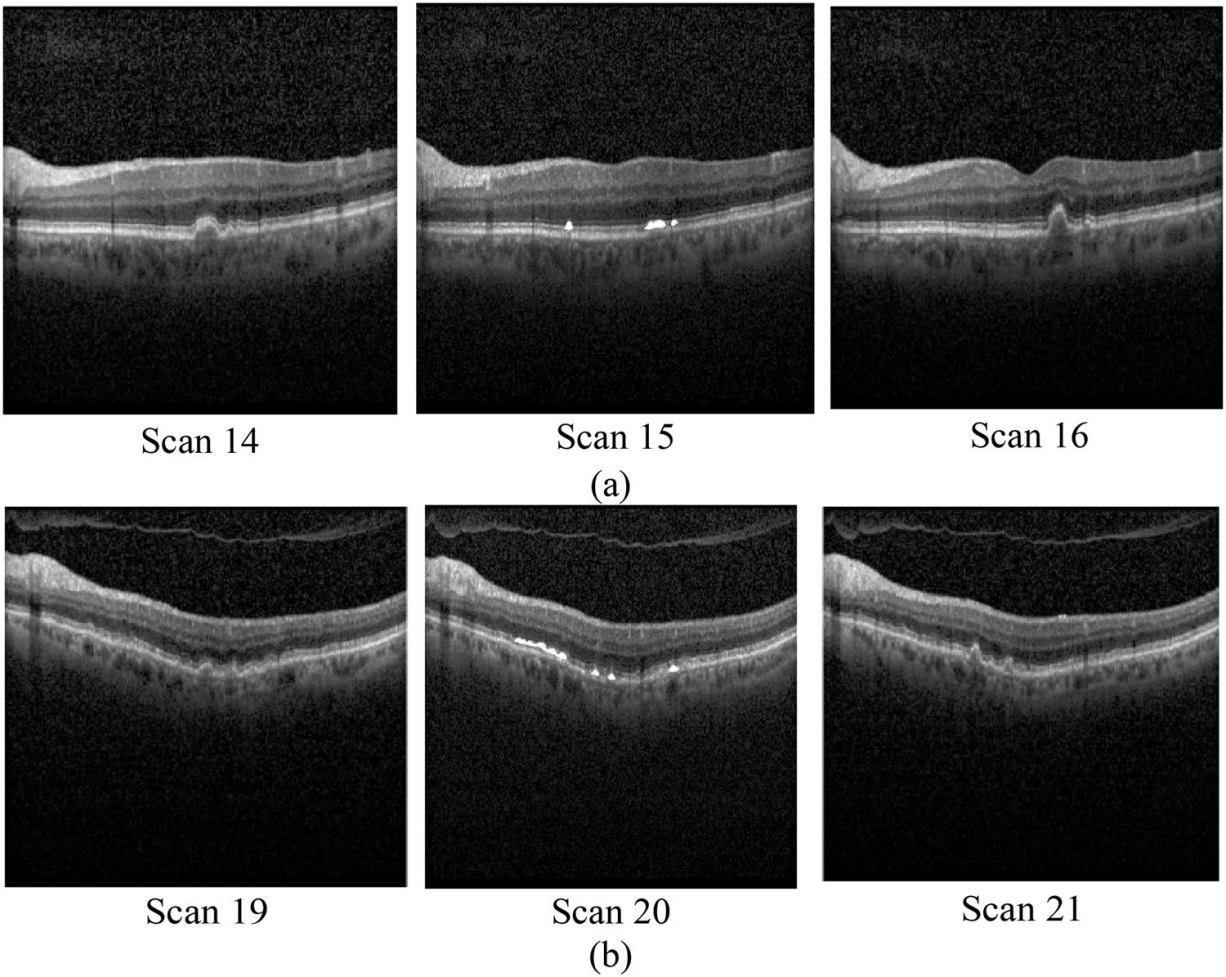
Part (**a**) Three consecutive B-scans with numbers 14, 15, 16, (**b**) three consecutive B-scans with numbers 19, 20, 21. White areas indicate cystic regions in Scan 15 and 20.

Figure 7 presents several consecutive OCT B-scans of a patient (in Dataset 1) which are correctly identified as cystic B-scans by our proposed HMM. Although the size of the cysts in the B-scans of Fig. 7 is small, the proposed model has correctly detected them. This is while SVM classifier has falsely detected some of them as non-cystic B-scans. For instance, SVM classifier has falsely detected Scan 15 and Scan 20 as non-cystic B-scans. The reason of correct identification by our proposed model is that these cystic images have been located after the images with big cysts in the sequence of OCT B-scans. Thus, the model can identify them as cystic images based on the probability of having a cystic image after a cystic image. This is one of the advantages of the proposed HMM compared to SVM classifier.

**Figure 8:**
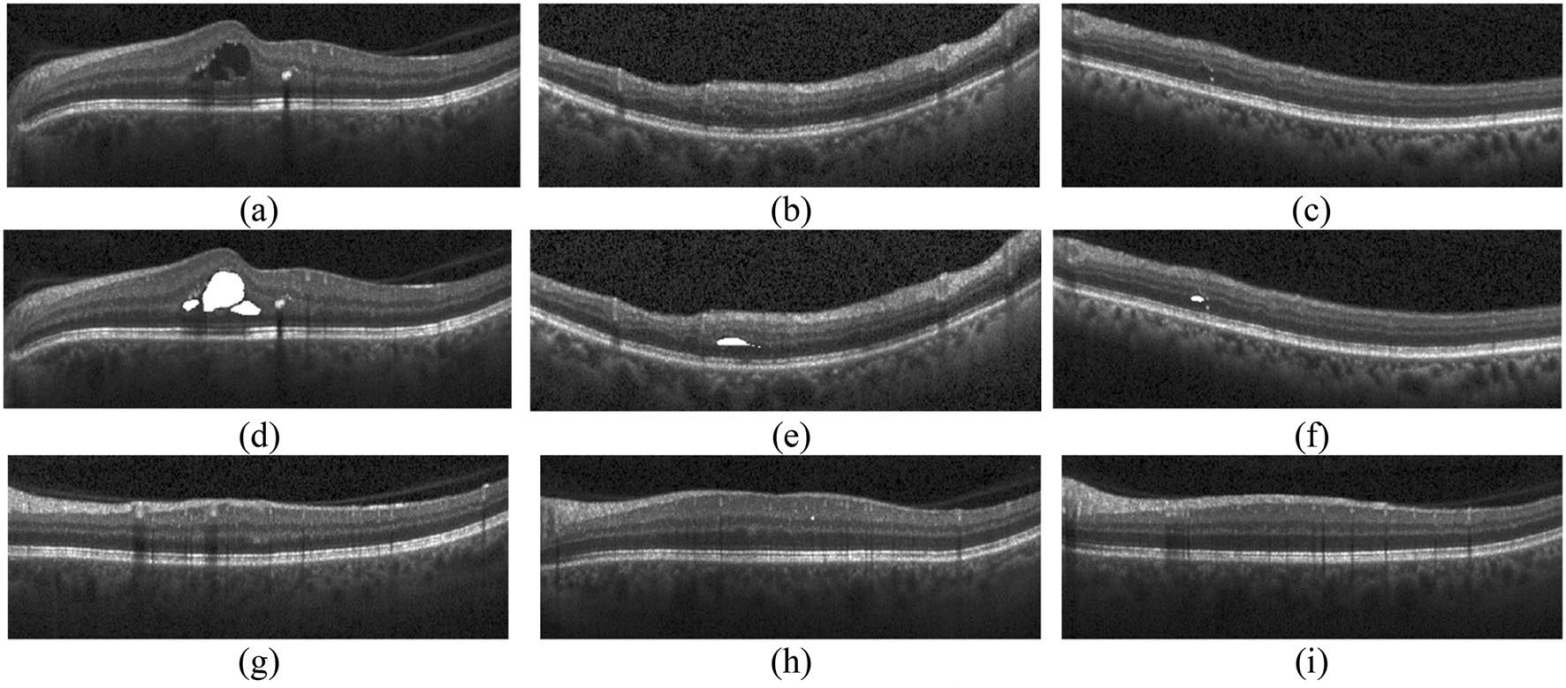
Some correctly identified figures by the proposed HMM. White areas in the second row present the cystic regions.

Figure 8 presents several OCT B-scans of Dataset 2 which are correctly identified by the proposed model. These images are wrongly identified by the method of Ref.^33^. It should be noted that parts (a), (b), and (c) are the images which include cysts and are correctly identified as cystic images. The labeled regions by the ophthalmologists for parts (a), (b), and (c) are presented in parts (d), (e) and (f), respectively. It should be noticed that parts (a) and (b) are correctly identified as cystic images by Ref.^42^ while this method detects part (c) as a non-cystic image. The reason is that the size of cyst in part (c) is very small. Also, parts (g), (h) and (i) are the ones do not contain any cyst and correctly detected as non-cystic by the proposed model while they are wrongly identified as cystic by Ref.^33^. It should be also mentioned that parts (h) and (i) are correctly identified by Ref.^42^, while part (g) is wrongly detected as a cystic image due to the existence of shadow in the image.

From speed point of view, it should be mentioned that the execution time for evaluating SVM classifier is equal to 0.3744 s while that of the proposed HMM is 0.2572 s. Moreover, it is very smaller than the method of Ref.^42^ which needs 6 s for processing. In addition, the sensitivity, specificity and accuracy of the proposed HMM are considerably higher than others. In fact, the proposed HMM outperforms the existing methods in terms of accuracy and execution time.

## Discussion

In the present study, we proposed a novel method for identifying the retinal OCT B-scans which consist of cysts. We focus on the identification of images containing cyst and not localization or segmentation purposes. In fact, there are applications where the localization or segmentation purposes are not important and only the detection of cystic images is sufficient. We believe that the most important application of our method is to automatically select the most informative B-scans among a large number of B-scans for ophthalmologists. In this way, the probability of error due to manual and time-consuming verifications is reduced.

In this research work, we test the power of different features in the discrimination of cystic images from images without cyst. In order to do so, we extract features using Harris, SURF, KAZE, MinEigen, BRISK, FAST, HOG, AlexNet methods and consider them as the inputs of an SVM classifier for classification. The results indicate that the accuracy of classification for the features extracted by AlexNet is higher than other methods. Also, the extracted features are fed to other classifiers including KNN and the same result is obtained.

**Figure 9:**
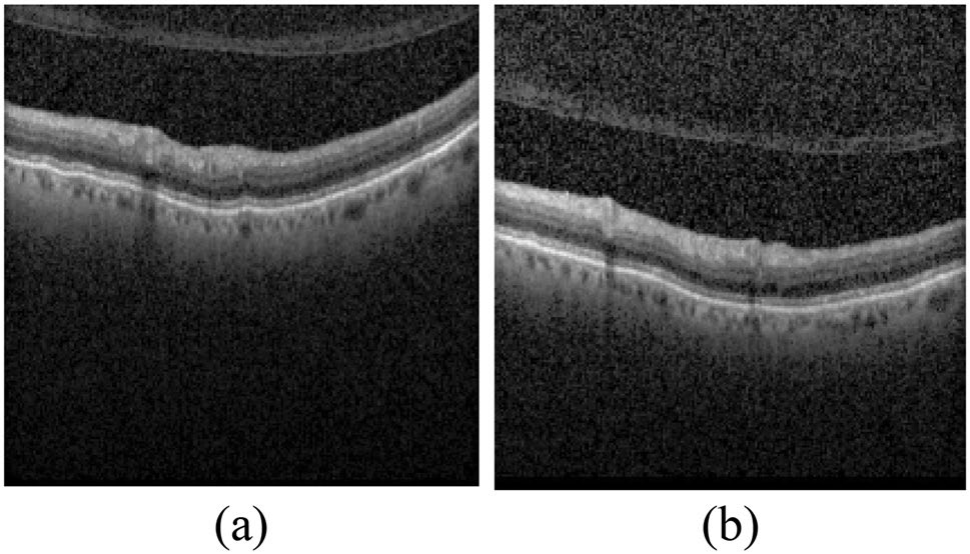
Two OCT B-scans mistakenly considered as cystic.

As obvious in Table 2, after the feature extracted by AlexNet, HOG is the feature capable of providing the highest accuracy. However, Fig. 9 presents two B-scans which are mistakenly classified as cystic when the HMM is trained using HOG feature. This is while the HMM correctly classifies them as without cyst, when it is trained by the feature extracted by AlexNet.

**Figure 10:**
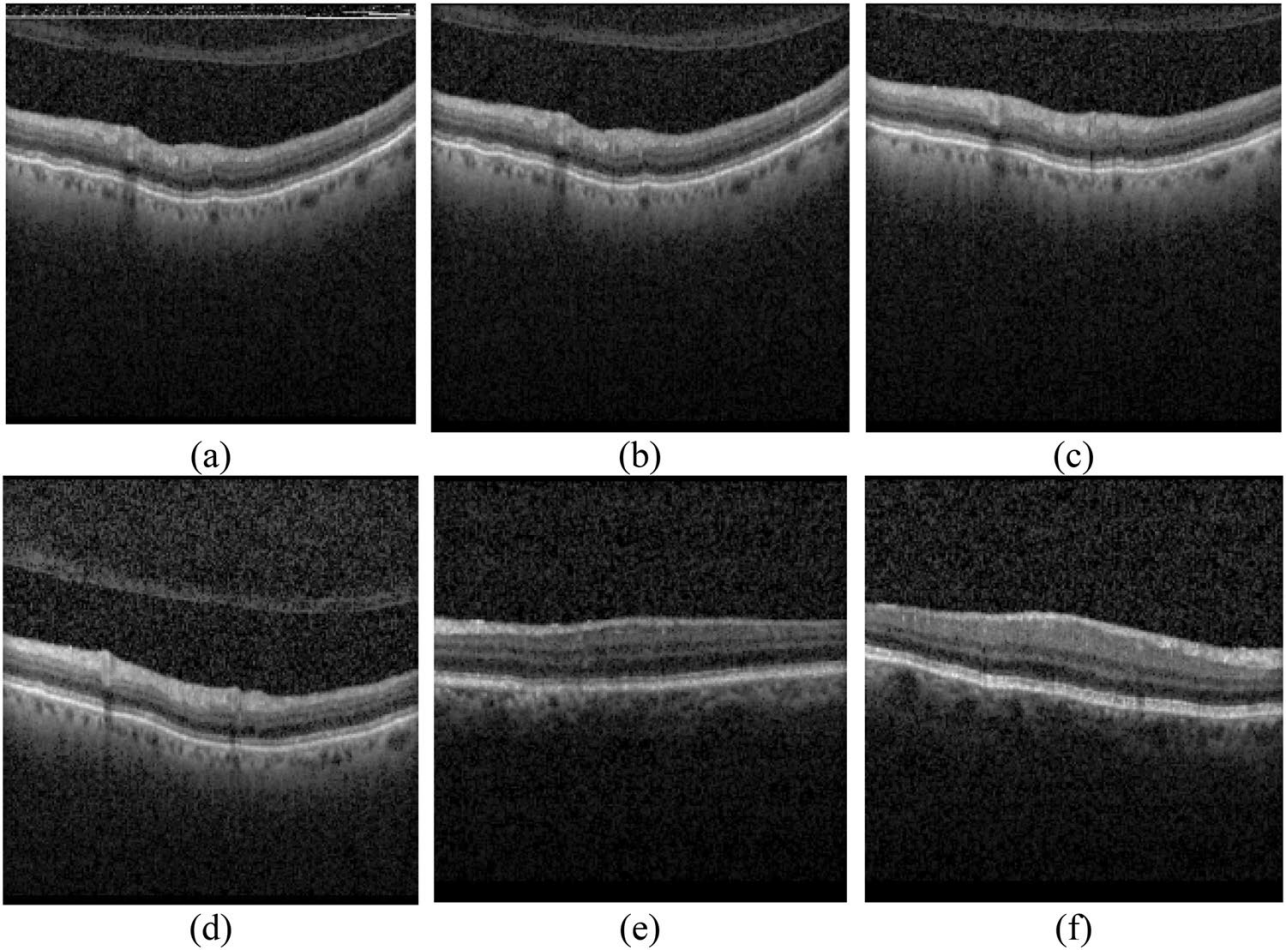
Several B-scans mistakenly identified as cystic by the proposed HMM.

There are also B-scans which do not include any cyst while they are mistakenly considered as cystic ones by the proposed HMM (when trained by AlexNet features). These B-scans are presented in Fig. 10. There are two reasons which may lead to this wrong detection. The first reason is that the curvature of RPE layer boundary in some images leads to a feature vector which is similar to that of a cystic image. This is true for parts (a), (b), (c) and (d). The second reason is that some B-scans are located after the B-scans with large cysts and if the probability of transfer from a cystic image to a cystic image (*a*_*11*_) is high, this wrong detection occurs. This reason is true for parts (e) and (f).

With respect to decoding of HMM with Viterbi algorithm explained in “Decoding HMM”, it is possible to have a case where the values of ${\updelta }_{t}\left(j\right)$ are the same and different states can be chosen with the same probability. This can lead to the failure of the model. However, the probability of having such a case is very small.

In future, we aim to extend the proposed model for the localization or even the segmentation of cysts in each image. The idea is that the existence of cyst in a region of an OCT B-scan depends on its neighbor regions. For instance, we divide an OCT B-scan to square patches. If there is a cyst or part of a cyst in a patch, there is the complementary part of cyst in the neighbor patch with high probability. Also, the existence of cyst in a patch can be considered as a hidden state which can produce observable features. Therefore, an HMM can determine the status of each patch from cyst point of view.

## Conclusions

In this paper a new method is proposed for the identification of retinal OCT B-scans including cysts. This method firstly evaluates the performance of several features in the discrimination of the images containing cysts from the others. For this purpose, a SVM classifier is utilized to classify the images to cystic and non-cystic images using different local features such as KAZE, SURF, Harris, HOG, FAST, MinEigen. Also, a feature extracted by AlexNet deep network by transfer learning method is also used. It is shown that among the local features and the feature extracted by AlexNet, the last one has the highest power in distinguishing cystic images from non-cystic ones. On the other hand, the existence of cyst in OCT B-scan can be considered as a hidden state which can produce some observable features. Therefore, the identification of cysts in the OCT B-scans can be modelled with a HMM the hidden states of which are the existence or absence of cysts. Also, the existence of cysts in an OCT B-scan of one patient depends on the existence of cyst in the previous B-scan. The parameters of such a model can be estimated through the extracted observation vectors from training samples. It is presented that the proposed model provides 97%, 93%, 95% for specificity, sensitivity and accuracy parameters, respectively. It is also capable of identifying the OCT B-scans including small cysts using the probability of having a cystic image after a cystic image.

## Data Availability

The datasets generated and/or analyzed during the current study are available in the following links. http://people.duke.edu/~sf59/Chiu_BOE_2014_dataset.htm; https://misp.mui.ac.ir.
